# None Too Much Quarantine

**Published:** 1885-10

**Authors:** 


### NONE TOO MUCH QUARANTINE.

Under the heading of "Modification of the Quarantine Needed," there appeared an editorial in the weekly issue of the *National Live Stock Journal*, arguing that the States west of the Mississippi had no longer good reason for continuing a strict quarantine against cattle from the States east of that river in which pleuro-pneumonia still exists, especially the States of Kentucky and Illinois. The authorities of the western ranging States must be fully aware that should the dreaded lung plague once reach the free cattle of their ranges, it cannot be stamped out, even with the possible hopes of final extinction which exist under the more restricted manner of herding cattle in the States East of the Mississippi.

The argument that the newly-appointed Live Stock Commission of Illinois will be found capable of confining any existing disease to the infested localities will not hold good until the ability of said Commission to do so has been proven by experience.

Two years ago no one thought it probable that the lung plague would extend its ravages over the Alleghanies to the cattle of Ohio, Illinois and Kentucky. Yet it did, and as it has once extended west of the Mississippi, there is no reason to assume that it may not again, under existing circumstances. The lung plague cannot be stamped out, nor the danger of its extension completely annulled so long as the State in which it exists adhere to their irrational manner of insufficiently remunerating owners for cattle slaughtered on account of the disease. Their action in this regard makes it necessary for each owner of infected or suspected herds to consider his own pocket before his responsibility to the good of all owners. Again, the lung plague cannot be stamped out until in the place of Cattle Commissions we have one State veterinarian, county and district veterinarians, and the same laws for the treatment of contagious animal diseases in every State.

Hence it is that the States west of the Mississippi must still adhere to their quarantine restrictions against Eastern cattle. The danger that threatens them is too great to allow of their amelioration under the impotent powers still existing in eastern States for the restriction of these diseases.

It is only by an obstinate adherence to this principle by the great breeding States that we can ever entertain hopes of finally bringing legislators, both National and State, to realize that these questions should be treated in a liberal and intelligent manner.

